# A strategy for quality control of ginkgo biloba preparations based on UPLC fingerprint analysis and multi-component separation combined with quantitative analysis

**DOI:** 10.1186/s13020-022-00618-3

**Published:** 2022-06-16

**Authors:** Li-na Liu, Hong-yu Jin, Zan Ke, Wei-yi Xu, Lei Sun, Shuang-cheng Ma

**Affiliations:** 1 Institute for Control of Chinese Traditional Medicine and Ethnic Medicine, National Institutes for Food and Drug Control, Beijing, 102629 People’s Republic of China; 2 Institute for cosmetic control, National Institutes for Food and Drug Control, 31 Huatuo Road, Daxing District, Beijing, 102629 People’s Republic of China

**Keywords:** Ginkgo biloba preparation, UPLC, Dietary supplement, Fingerprint, Quantitative analysis, Traditional Chinese medicine, Chemometric analysis, Organic acids, Quantitative analysis of multi-components, Quality evaluation

## Abstract

**Background:**

Many studies have assessed the fingerprint and quantitative analysis of Ginkgo biloba preparations, but the fingerprint mainly focuses on flavonoid glycosides. However, according to our previous study, the differences among diverse manufacturers mainly involve organic acids.

**Methods:**

A novel reverse-phase liquid chromatography assay using diode array detection was developed for evaluating Ginkgo biloba preparations for quality based on a chromatographic fingerprint allowing the simultaneous assessment of eleven compounds, including four organic acids, six flavonol glycosides and one flavonoid aglycone. And the method was applied to 51 batches of Ginkgo biloba preparations from manufacturers in China. Chemometric approaches were performed for evaluating 51 batches of Ginkgo biloba preparations from various manufacturers.

**Results:**

The similarity values among the chromatograms of 51 samples ranged from 0.45 to 1.00, showing that the quality of Ginkgo biloba preparations produced by different manufacturers varied greatly. Data analysis of the 51 batches of GBP samples suggested significant variations of the total contents of all 11 targets, also demonstrating the quality difference of GBP samples. There were significant differences in organic acids in particular.

**Conclusion:**

Combining the chemical fingerprint and quantitative assessment revealed significant variations in the examined commercial products with regard to organic acids. Thus, this study provided a more comprehensive tool for monitoring the quality consistency of Ginkgo biloba preparations.

## Introduction

Ginkgo biloba has been named a living fossilas one of the oldest tree species in the world; it is widely distributed in China. The therapeutic activities of Ginkgo biloba leaves have been well-known in traditional Chinese medicine for millennia. Ginkgo biloba leaf extract (GbE) was introduced into the medical practice in 1965 by Dr. Willmar-Schwabe company. The product treats multiple signs of early cognitive impairment and severe dementia due to primary degenerative, vascular and mixed factors, besides peripheral arterial occlusive pathologies and diverse neurosensory disorders [1]. This extract comprises flavonol glycoside (24%), terpene lactone (6%), proanthocyanidin(7.0%) and organic acid (13%) constituents, with flavonol glycosides and terpene lactones representing the major and bioactivecomponents that have been used as quality indicators of GbE and preparation steps [2].

Flavonoids are potent antioxidants and free radical scavengers [3]. Since there are many flavonol glycosides in GbE, the reference materials are difficult and costly to separate. Flavonol standardization in GbE and preparations is currently carried out by determining total flavonol glycoside amounts from aglycone levels in acid-hydrolyzed specimens. After aglycones, including quercetin, kaempferol and isorhamnetin, undergo acid hydrolysis and HPLC, further analysis could readily estimate the actual total amounts of flavonol glycosides. However, since the determination is not the prototype of flavonol glycosides, GbE adulteration with flavonol aglycones or glycosides, as free or bound components cannot be ruled out [4, 5]. In order to prevent the illegal addition of rutin and other cheap and readily available flavonol glycosides to improve the content of flavonoid aglycones, inspection items for flavonoid aglycones have been formulated, but the inspection of peak area ratios of flavonoid aglycones could not stop the illegal addition or falsification of Ginkgo biloba extract. Profiling genuine flavonol glycosides could help detect adulteration. In the 2020 edition of the Chinese Pharmacopoeia [6], a fingerprint item was added to the standard of Ginkgo biloba extract.

Organic acids are common chemical components in a class of plants with overt biological activities. There are 13% carboxylic acids in Ginkgo biloba preparations, including quinic, shikimic, gallic, protocatechuic, caffeic, 6-hydroxybenzoic and p-hydroxybenzoic acids [7–10], Of these, protocatechuic and 6-hydroxykynurenic acids are potent scavengers of authentic ONOO-[11]. However, carboxylic acids have not received much attention with regard to their pharmacological properties.

Many studies have assessed the fingerprint and quantitative analysis of GbE [12, 13], but the fingerprint mainly focuses on flavonoid glycosides. However, according to our previous study, the differences among diverse manufacturers mainly involve organic acids. A previous study [14] reported that the content of flavonoid aglycones is increased due to unauthorized changes in the extraction process for Ginkgo biloba extract and illegal addition of quercetin. The United States Pharmacopoeia also limits free quercetin content [15]. Therefore, the content of flavonoid aglycones also deserves attention. In order to compare quality differences among various manufacturers, chromatographic fingerprint analysis should not only pay attention to flavonoid glycosides, but also to organic acids and flavonoid aglycones. In this study, we combined the chemometric analysis of the chromatographic fingerprint and concurrent detection of its main biologically active constituents, including organic acids, flavonoid glycosides and free flavonoids, for assessing Ginkgo biloba preparations (GBP) for quality. In addition, we examined the quality differences of GBP from various manufacturers.

## Materials and methods

### Samples and reagents

HPLC-grade methanol was provided by Fisher Scientific (USA). MS-grade phosphoric acid was from Aladdin (China). Ultrapure water was prepared with a Milli-Q system (Millipore, USA). The remaining reagents, of analytical grade, were provided by Beijing Chemical Factory (China). The reference standards of three organic acids, including gallic acid (S1, GA), protocatechuic acid (S2, PA) and p-hydroxybenzoic acid (S3, pHA), two flavonol glycosides, including lutin (S4) and isorhamnetin 3-rutinoside (S5, IR), and one flavonoid aglycone (quercetin, S6) were obtained from the National Institutes for Food and Drug Control (NIFDC) in China. 6-Hydroxykynurenic acid (S7, 6-HKA) was provided by Zhejiang Kangenbei Pharmaceutical (China). Reference standards of 4 flavonol glycosides, including quercetin 3-O-(2″, 6″-α-l-dirhamnopyranosyl)-β-d-glucoside (S8, QDG), kaempferol-3-O-rutinoside (S9, KR), quercetin-3-O-α-l-rhamnopyranocyl-2″-(6‴-p-coumaroyl)-β-d-glucoside (S10, QCG) and kaempferol-3-O-α-l-rhamnopyranocyl-2″-(6‴-p-coumaroyl)-β-d-glucoside (S11, KRCG), were purchased from Shanghai Yuanye biological technology (China). The purity of the standard compounds obtained from NIFDC was more than 90%, as determined by the mass balance. The purity of other standard compounds was higher than 90%, as determined by HPLC.

Fifty-one different GBP samples were purchased from the market in China. All fifty-one samples were in the solid form (tablets and capsules), and were analyzed before the respective expiration dates. Commercial products, including GBE-A (lot no. M17253), GBE-B (0611517), GBE-C (20181003), GBE-D (1811081), GBE-E (180601), GBE-F (181201 and 181002), GBE-G (1852018, 1852022, 1852024 and 1862028), GBE-H (180404), GBE-I (180828), GBE-J (180401), GBE-K (1901033, 1901023, 180929, 181119 and 1806162), GBE-L (181101 and 180706), GBE-M (180801), GBE-N (1804941 and 1901947), GBE-O (180702), GBE-P (180313), GBE-Q (20180415, 20180805 and 20181112), GBE-R (A1806007, A1812003, and A1902004), GBE-S (E17110104 and E17120104), GBE-T (180201), GBE-U (1901001), GBE-V (181208, 180604 and 181012), GBE-W (18102223, 18,102321 and 18100521), GBE-X (171214, 180916, 181023, 180314, 180615, 181216 and 190402), and GBE-Y (02A180152 and 02J181252), were produced by different manufacturers. GBE-X (lot no. 190402) was utilized for chromatography conditions and assay validation.

### Instruments

UPLC was performed on a Waters Acquity UPLC system consisting of a Binary high-pressure pump with vacuum degasser, a diode-array detector (DAD), an autosampler and the Acquity UPLC^®^ HSS T3 column (2.1 × 100 mm with 1.7-μm internal diameter; Waters, USA).

### Chromatography conditions

The separation was performed under the following conditions: column temperature, 40 ℃; injection volume, 1μL; flow rate, 0.4 mL/min. Mobile phases A and B were 0.2% phosphoric acid in water and 0.2% phosphoric acid in acetonitrile (both v/v), respectively. Elution in UPLC used the following linear gradient: 0–5 min, 5–10% B; 5–5.01 min, 10–16.6% B; 5.01–9.6 min, 16.6–19.8% B; 9.6–14 min, 19.7–24% B; 14–16 min, 24–27% B; 16–20 min, 27–30% B; 20–25 min, 30–80% B; 25–25.01 min, 80–5% B; 25.01–30 min, 5% B. Monitoring was performed at 260 nm.

### Data analysis

Raw data were analyzed and processed with Waters Empower Chemstation. Chromatograms, exported as.cdf files and assessed for fingerprint with ‘ChemPattern edition 2.0’ (Chemmind Technologies, China). Principal component (PCA) and cluster (CA) analyses were carried out with 51 batches of samples. Additional clustering parameters included distance measurement, cosine, linkage method and nearest neighbor.

### Standard and sample solution preparations

Stocks of reference standards were made in methanol/water (v/v 8:2) and kept at 4 °C shielded from light until use. Afterwards, adequate amounts of these stocks were diluted using methanol–water (v/v 8:2) to prepare working solutions at the desired concentrations.

Totally 10 tablets or capsules were weighed and finely powdered. The coating was first removed from sugar-coated tablets. An equivalent of about 40 mg of Ginkgo extract powder was transferred to a volumetric flask, and 20 mL of methanol–water (v/v 8:2) was supplemented. Mixing was performed by ultrasonication for 20 min after sample weighing. The mixture was allowed to cool down, followed by another weighing; the lost weight was replenished with methanol–water (v/v 8:2). After mixing well and filtering (0.22 μm), the resulting filtrate was analyzed.

## Results

### Optimization of the extraction process

Extraction optimization aimed to allow a comprehensive analysis of the components of Ginkgo biloba extract preparations. Different extraction solvents (methanol concentration in water, at v/v 50:50, v/v 80:20 and 100:0), durations (20, 30 and 40 min), and material-to-liquid ratios (1:250, 1:500 and 1:1000) were examined. As a result, extraction with methanol–water (80:20, v/v) was most efficient with the highest number of chromatographic peaks. A solid-to-liquid ratio of 1:500 and an extraction duration of 20 min were also the best conditions.

### Detection wavelength selection

Choosing the adequate wavelengths is very important for the accurate determination of the eleven compounds and deriving a great amount of information. UV spectra for the constituents had peaks at 260 and 360 nm. Since there were more peaks at 260 nm compared with 360 nm, 260 nm was selected as wavelength for fingerprinting.

### Method validation

To assess whether the fingerprint analysis is applicable, method precision, reproducibility and stability were tested. Precision was examined via 6 consecutive injections of a specimen and the assessment of all fingerprint peaks; precision was reflected by relative standard deviations (RSDs) of retention time (RT) and peak area (PA) not exceeding 0.44% and 2.30%, respectively. Reproducibility was examined by analyzing 6 specimens prepared from one sample, and was reflected by RSDs of RT and RA not exceeding 0.62% and 3.20%, respectively. Stability was evaluated by testing a single specimen at 4-h intervals within a 24-h period at ambient; RSDs of RT and RA were below 0.51% and 2.88%, respectively, indicating stability. The above findings showed the novel chromatographic assay is reliable for GBP fingerprint assessment.

To assess the value of the new UPLC assay in quantitating the eleven constituents, its linearity, limit of detection (LOD), limit of quantification (LOQ) and accuracy were further determined. Duplicate injections were averaged. The LOD and LOQ were determined by successively reducing the standard’s level until the least detectable peak. This level was multiplied by 3 and 10 to derive LOD and LOQ values, which were 0.005–0.26 ng and 0.015–0.87 ng, respectively. RSDs for precision and stability were below 1.0% and 2.0%, respectively. All calibration data are found in Table 1. Peak areas (y-axis) and concentrations (x-axis) showed a good correlation (R^2^ > 0.995) for each compound for the examined concentrations at respective wavelengths; the linear range of each compound was wide enough for use, with a 30 times ratio between the high and low concentration limits. Quantification accuracy was assessed by the recovery test and calculated based on the standard addition technique. Recovery rates for the 11 compounds were in the range of 97.5%–105%, and RSDs were below 3.4%. These data indicated the 11 constituents could be accurately measured at the same time by the novel assay. The results of the quantitative analysis for each compound are shown in Table 1.

**Table 1:** Regression equation, precision, repeatability, stability, sensitivity and recovery of the eight compounds

Reference Substance	Regression Equation	R2	Linear Range/ng	Precision	Repeatability	Stability	Recovery		LOD(ng)	LOQ(ng)
				RSD%	RSD%	RSD%	%	RSD%		
6-HKA	y = 43,222 x—116	1.000	0.2–6	0.36	3.57	0.51	105	3.00	0.02	0.07
pHA	y = 19,127 x—240	1.000	0.11–3.3	0.70	2.90	1.21	101	3.44	0.01	0.03
GA	y = 9963 x + 1377	0.995	0.017–0.51	0.22	4.36	0.84	98.9	2.15	0.005	0.015
PA	y = 106,127 x—2,445	1.000	0.45–13.5	0.21	2.77	0.59	107	2.77	0.03	0.09
KR	y = 233,564 x—964	1.000	5–150	0.26	3.49	0.76	97.5	2.44	0.12	0.40
KRCG	y = 590,965 x—1,866	1.000	6.9–207	0.40	3.34	1.12	103	3.20	0.07	0.20
QRGR	y = 201,989 x—105	1.000	5.4–162	0.32	3.61	1.05	102.7	1.85	0.1	0.35
QCG	y = 327,385 x—1,535	1.000	7.7–331	0.24	3.22	0.72	98.1	2.90	0.16	0.52
lutin	y = 204,300 x—756	1.000	3.6–108	0.35	3.35	1.03	104	2.82	0.01	0.03
IR	y = 121,021 x—1,054	1.000	4.5–135	0.29	2.77	0.95	102	1.72	0.26	0.87
quercetin	y = 52,557 x + 112	1.000	1.13–33	0.34	3.49	1.11	105	2.46	0.07	0.24

### Quantitative analysis

The new assay was successfully utilized to simultaneously detect eleven active ingredients for examining quality differences among 51 GBP batches. Chromatogram peaks were identified via comparison of RTs and online UV spectra with those of reference compounds. To facilitate the comparison of the amounts of various constituents in distinct specifications, we normalized the contents to values in a 40-mg extract. Table 2 summarizes the contents of these eleven compounds in 51 GBP specimens. The results revealed the eleven analytes showed significantly diversified amounts among specimens. The contents of the analytes varied substantially among specimens. Most of the time, KR, rutin, QDG and IR constituted the major components, whose average levels were above 0.7 mg per 40 mg GBE. Meanwhile, 6-HKA and GA were below the detection limit in some batches. The contents of 6-HKA, GA, PA and quercetin in the 51 batches varied greatly, with RSD above 80%. The contents of KR, KRCG, QDG, QCG and IR in different batches varied relatively less, with RSDs no more than 30%. The total contents of the major bioactive components amounted to 6.36 mg per 40 mg GBE, in the range of 4.523 to 9.727 and the RSD was 19.8%. There was a certain correlation among the contents of the three organic acids. The correlation coefficient for pHA and GA was 0.72; that for GA and PA was 0.85, and the correlation coefficient for pHA and PA was 0.71.

**Table 2:** The results of content determination (mg per 40 mg GBE) and similarity analysis

Sample	Content (mg/40 mg GBE)												Similarity
	6-HKA	pHA	GA	PA	KR	KGCG	QDG	QCG	Lutin	IR	Quercetin	Total	
A1	0.048	0.033	0.014	0.365	0.818	0.820	1.009	0.886	2.075	0.644	0.038	6.749	1.00
B1	0.077	0.051	0.053	0.715	0.814	0.786	1.070	0.885	2.116	0.632	0.101	7.298	0.93
C1	0.007	0.016	0.003	0.070	0.924	1.040	1.080	1.240	2.196	0.844	0.091	7.511	0.89
D1	0.020	0.025	0.005	0.141	0.756	0.947	0.675	1.194	0.774	0.751	0.056	5.343	0.90
E1	0.010	0.037	–	0.090	0.724	1.193	0.990	1.311	0.773	0.563	0.045	5.736	0.86
F1	0.022	0.061	0.065	0.908	0.831	0.913	1.117	1.111	1.964	0.762	0.023	7.779	0.86
F2	0.007	0.065	0.049	0.955	0.897	1.007	1.188	1.215	1.071	0.835	0.027	7.317	0.87
G1	0.007	0.026	–	0.213	0.791	0.692	0.886	0.856	0.805	0.786	0.018	5.080	0.92
G2	0.006	0.035	–	0.183	0.858	0.815	0.864	1.135	0.887	0.796	0.020	5.598	0.86
G3	0.008	0.031	–	0.187	0.803	0.803	0.885	1.119	0.869	0.741	0.024	5.471	0.86
G4	0.004	0.037	–	0.215	0.878	0.893	1.019	1.204	0.915	0.818	0.026	6.009	0.91
H1	0.007	0.026	–	0.068	0.744	1.101	1.116	1.384	0.809	0.704	0.038	5.997	0.86
I1	0.057	0.027	0.019	0.421	0.960	0.989	0.892	1.240	1.144	0.886	0.022	6.656	0.98
J1	0.029	0.074	0.047	0.692	0.925	1.042	1.206	1.292	0.859	0.833	0.022	7.020	0.93
K1	0.018	0.032	–	0.056	0.598	1.026	0.842	1.227	0.693	0.507	0.056	5.055	0.75
K2	0.018	0.035	–	0.062	0.627	1.011	0.831	1.206	0.700	0.536	0.052	5.078	0.86
K3	0.020	0.034	0.009	0.066	0.612	0.970	0.908	1.135	1.636	0.521	0.052	5.963	0.74
K4	0.019	0.034	–	0.032	0.613	1.003	0.961	1.173	1.675	0.526	0.048	6.086	0.73
K5	0.018	0.028	–	0.058	0.597	1.002	0.932	1.170	0.833	0.511	0.078	5.227	0.85
L1	0.009	0.079	0.054	1.132	0.880	1.026	1.127	1.301	0.823	0.835	0.021	7.288	0.83
L2	0.005	0.030	–	0.100	0.491	0.898	0.614	1.121	0.501	0.492	0.272	4.523	0.71
M1	0.009	0.022	–	0.105	1.011	1.324	1.152	1.559	0.973	0.947	0.048	7.150	0.82
N1	0.011	0.025	0.001	0.055	0.797	1.105	1.010	1.308	0.797	0.726	0.060	5.895	0.84
N2	0.014	0.027	–	0.067	0.808	1.071	0.829	1.240	0.790	0.735	0.053	5.634	0.87
O1	–	0.009	–	0.008	0.706	1.098	0.339	1.373	0.820	0.618	0.075	5.046	0.78
P1	0.016	0.034	0.002	0.200	1.108	1.034	1.056	1.124	1.207	1.182	0.072	7.035	0.88
Q1	0.009	0.039	0.002	0.142	0.859	0.875	0.650	1.112	0.800	0.717	0.030	5.233	0.90
Q2	0.023	0.038	0.010	0.195	0.708	0.932	0.910	1.244	1.873	0.593	0.018	6.545	0.92
Q3	0.004	0.035	0.005	0.112	0.773	0.840	0.735	1.119	1.725	0.654	0.042	6.043	0.86
R1	0.002	0.023	–	0.039	0.696	0.982	0.805	1.160	0.615	0.701	0.142	5.167	0.83
R2	0.008	0.029	–	0.037	0.752	1.071	0.943	1.256	0.669	0.758	0.148	5.672	0.73
R3	0.009	0.029	–	0.038	0.761	1.075	0.925	1.248	1.358	0.772	0.155	6.370	0.74
S1	0.034	0.050	0.030	0.697	1.296	1.160	0.935	1.404	2.906	1.185	0.029	9.727	0.96
S2	0.058	0.045	0.024	0.669	1.333	1.135	1.118	1.362	1.247	1.232	0.032	8.255	0.93
T1	0.002	0.027	–	0.063	0.700	1.053	0.904	1.207	0.630	0.708	0.151	5.445	0.71
U1	0.003	0.015	–	0.037	0.935	1.224	0.904	1.419	0.873	0.859	0.127	6.395	0.78
V1	0.029	0.046	–	0.049	0.913	1.496	1.440	1.749	2.507	0.790	0.082	9.101	0.45
V2	0.015	0.045	0.052	0.033	0.783	0.059	0.086	0.368	1.314	1.827	0.041	4.624	0.86
V3	0.005	0.023	–	0.022	0.818	0.896	0.724	1.070	0.920	0.759	0.036	5.272	0.84
W1	0.004	0.012	–	0.018	0.947	1.115	0.912	1.262	0.877	0.906	0.020	6.071	0.78
W2	0.003	0.013	–	0.018	0.961	1.135	0.981	1.319	1.090	0.923	0.022	6.465	0.86
W3	0.012	0.019	0.011	0.059	0.807	1.019	0.734	1.172	0.915	0.859	0.095	5.701	0.88
X1	0.035	0.032	0.020	0.554	0.809	1.123	1.107	1.363	0.819	0.756	0.021	6.638	0.83
X2	0.064	0.027	0.019	0.439	0.868	1.060	1.050	1.350	1.171	0.803	0.024	6.875	0.92
X3	0.050	0.025	0.016	0.367	0.713	1.005	0.712	1.407	2.190	0.730	0.019	7.234	0.95
X4	0.047	0.018	0.015	0.349	0.704	1.109	0.929	1.434	1.066	0.647	0.024	6.341	0.85
X5	0.039	0.020	0.034	0.651	1.064	1.614	1.204	2.104	1.132	1.018	0.025	8.905	0.93
X6	0.134	0.048	0.031	0.524	1.116	1.348	1.800	1.805	1.507	1.045	0.029	9.384	0.95
X7	0.056	0.028	0.016	0.425	0.793	1.031	1.042	1.357	1.139	0.766	0.021	6.673	0.95
Y1	0.002	0.028	–	0.028	0.665	1.105	1.090	1.285	0.896	0.543	0.048	5.690	0.73
Y2	0.003	0.033	–	0.031	0.656	0.936	0.753	1.318	0.808	0.627	0.074	5.237	0.53
min	–	0.009	–	0.008	0.491	0.059	0.086	0.368	0.501	0.492	0.018	4.523	
max	0.134	0.079	0.065	1.132	1.333	1.614	1.800	2.104	2.906	1.827	0.272	9.727	
average	0.022	0.033	0.012	0.250	0.824	1.020	0.941	1.253	1.172	0.783	0.057	6.365	
RSD	113.68%	44.27%	150.65%	113.99%	20.09%	21.11%	26.67%	19.53%	46.48%	28.98%	86.04%	18.98%	

The limit standard of quercetin was normalized to the 40 mg extract; the limit of quercetin in the supplementary test method was 0.4 mg per 40 mg extract, and its limit in the USP was 0.2 mg per 40 mg extract. From the determination results, the maximum content of quercetin was 0.272 mg per 40 mg extract, and the others’ amounts were all below 0.2 mg. The content of quercetin in one batch (sample L2) was significantly higher than in the other samples. It was worth paying attention to whether there were problems, including changing the extraction process without authorization and illegal addition of quercetin. The limit of quercetin in the supplementary test method is a relatively loose standard, which can be further tightened.

In order to reflect the quality fluctuations between different GBP batches, the parameter P was applied [16]. The closer the P value was to 100%, the better the consistency between different batches was. The formula for calculation was as follows:

$$\mathrm{P}=\frac{{\mathrm{C}}_{\mathrm{i}}}{\overline{{\mathrm{C}}_{\mathrm{i}}}}\times 100\mathrm{\%}$$

In this equation, Ci indicated the measured concentration of each component, while $\overline{{\mathrm{C}}_{\mathrm{i}}}$ indicated the average concentration in 51 batches of GBP.

**Fig. 1:**
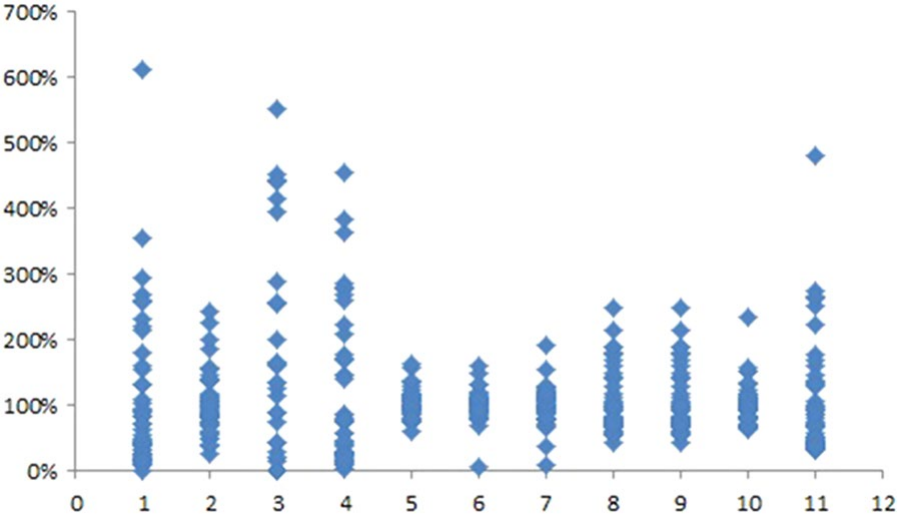
Differences in 51 batches of GBP (1.6-HKA, 2. pHA, 3.GA 4. PA, 5. KR, 6. KGCG 7. QDG, 8. QCG, 9. Lutin,10.IR,11. Quercetin)

The P values of the 11 components were shown in Fig. 1. As shown in the figure, the component GA, 6-HKA and PA varied greatly, and the max P value of 6-HKA can reach up to 611%, which indicated that the quality of different samples might fluctuate greatly. The flavonol glycosides component, including KR, KGCG, QDG, QCG, lutin and IR, P values showed the better consistency compared with organic acids, indicating a better consistency among samples in qualitative evaluation, but the P value still far beyond the considered acceptable range of 75–125% [16]. Therefore, more attention should be paid to study and normalize the content of organic acids for ensuring the quality of GBP.

### UPLC fingerprinting and similarity analysis (SA)

**Fig. 2:**
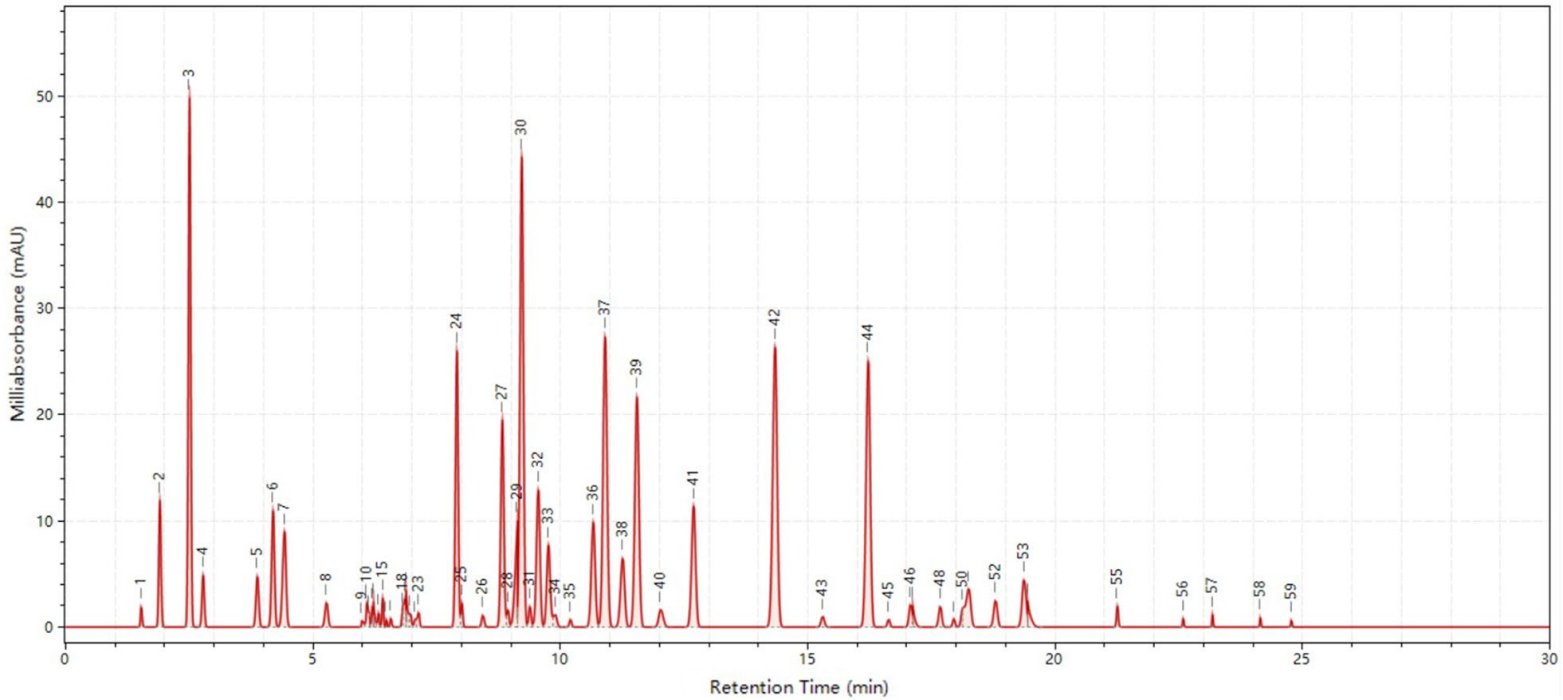
The fingerprint of common patterns of GBP(1.GA, 3.PA, 5. pHA, 6.6-HKA, 24.QDG, 30. Lutin, 37.KR, 39. IR, 42. QCG, 43. Quercetin, 44.KRCG)

**Fig. 3:**
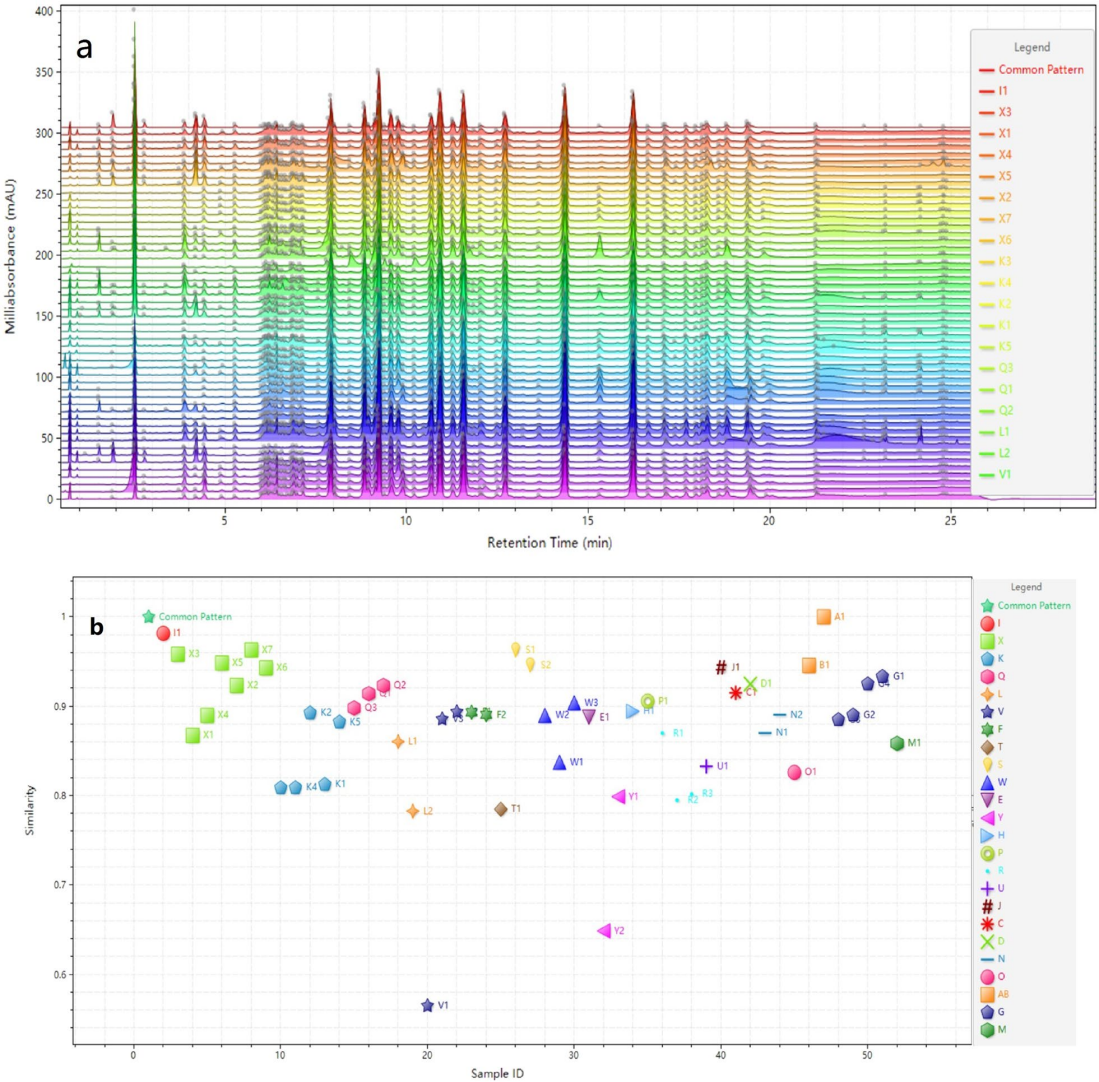
Chromatographic fingerprints (**a**) and similarity results (**b**) of 51 batches of GBP

The fingerprints of the 51 batches of GBP were analyzed. UPLC chromatograms for all 51 specimens were converted to.cdf files and imported into the ‘ChemPattern edition 2.0’ for further data processing. SA was carried out according to the correlation coefficients of raw data. The similarity values (Table 2) of the chromatograms of the 51 samples ranged from 0.45 to 1.00 (Fig. 3), showing that the quality of GBP produced by different manufacturers varied greatly. Samples with similarity values below 0.85 were collected and further assessed (Table 3). In this study, samples were collected from 25 manufacturers, and the similarity values of samples produced by 10 manufacturers were lower than 0.85, and the similarity values of more than two batches from some manufacturers were all lower than 0.85 ( manufacturer codes were L, R and Y). Overall, 59 commonly detected peaks were found in the reference fingerprint chromatogram (Fig. 2), of which 11 were successfully identified.

**Table 3:** Statistical table of low similarity samples

Manufacturer code	Low similarity batch	Total batch	Low similarity ratio(%)
K	3	5	60
L	2	2	100
M	1	1	100
N	1	2	50
O	1	1	100
R	3	3	100
T	1	1	100
V	2	3	67
W	1	3	33
Y	2	2	100

### Hierarchical cluster analysis (HCA) of fingerprints

**Fig. 4:**
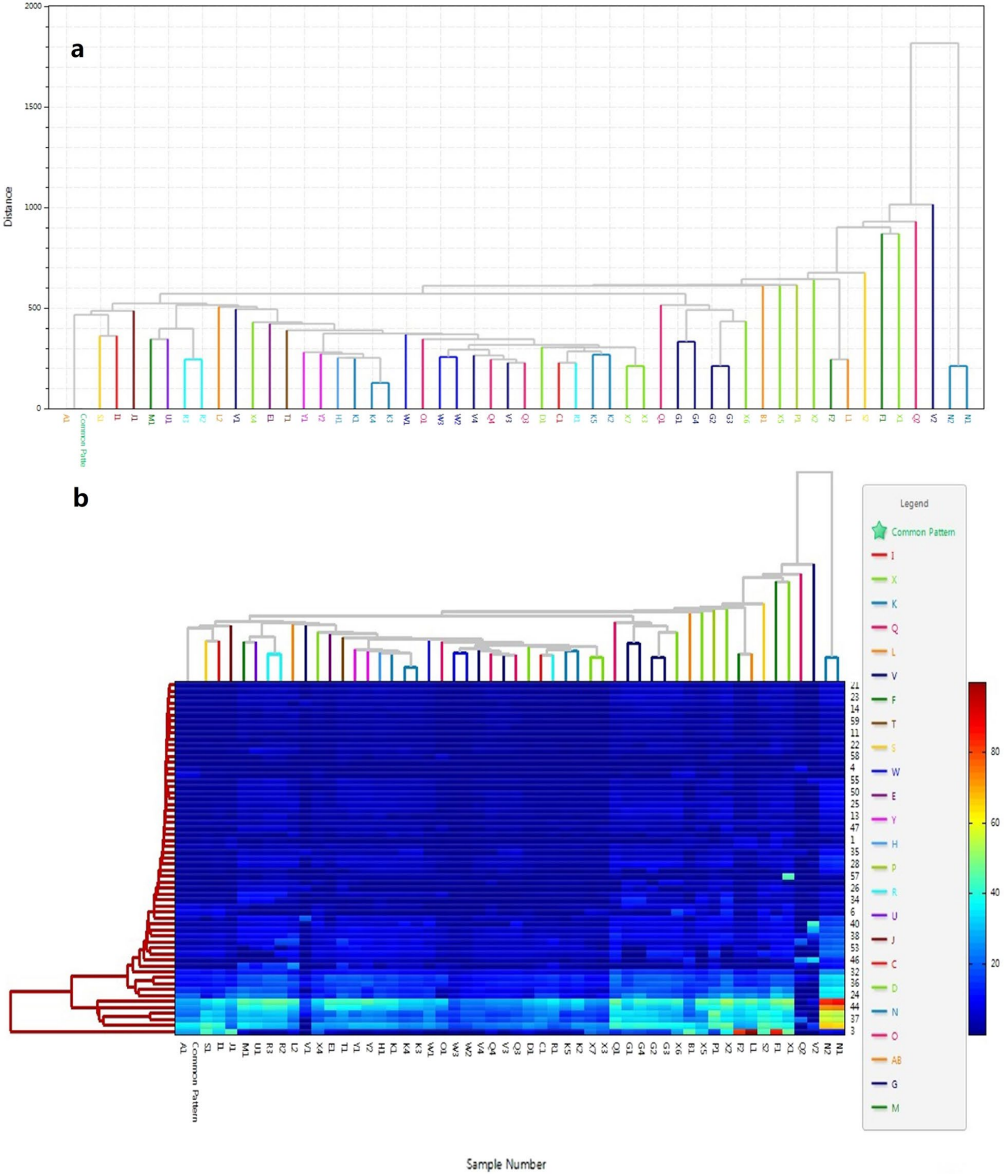
Cluster analysis tree map (**a**) and heat map (**b**) for the 51 GBP batches

HCA represents a multivariable analysis technique displaying complex raw data visually and achieving the classification of test specimens. HCA was used to assess evaluation data of fingerprints derived from UPLC. Tree diagrams are generally utilized for depicting similarity and dissimilarity among samples, respectively [17]. HCA was used to group GBP into different categories to further classify the GBP samples of different quality levels. Hierarchical cluster analysis was carried out with the ‘ChemPattern edition 2.0’ software. Cluster analysis tree map and cluster analysis heat map are shown Fig. 4. As shown in the figure, many samples produced by the same manufacturer can not be clustered together well, while some products from different manufacturers could be grouped into one category. The product clustering of different manufacturers was not obvious. Samples N1 and N2 were far away from other samples.

### Principle component analysis (PCA) of fingerprints

PCA represents a multivariable statistical analysis technique combining many variables to generate a new and small set of independent comprehensive variables through linear transformation. PCA retains the major information in large amounts of variables, reducing data dimension, and is commonly applied [15, 18].

**Fig. 5:**
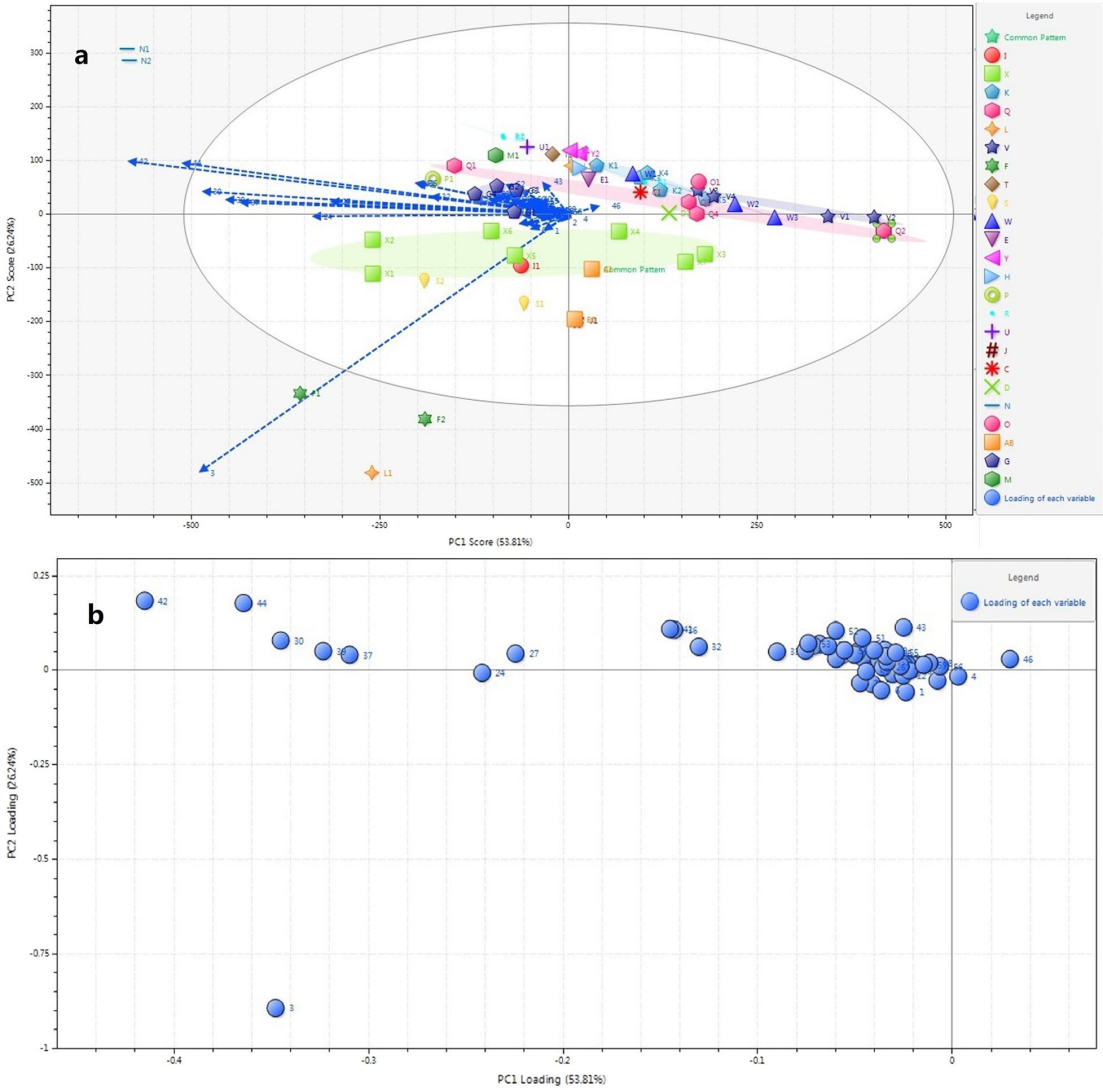
PCA score scatter plot (**a**) and loading scatter (**b**) of GBP

The main component analysis was carried out after sample determination data was standardized with the Chempattern software, as shown in Fig. 5. The contribution rates of the first (PC1) and second (PC2) principal components were 53.81% and 26.20%, respectively, with a cumulative value of 80.01%. This indicated that PCA can reflect the differences among samples more comprehensively. The PCA’s scatter diagram showed that most samples from different manufacturers had no obvious clustering trend, only some samples from different manufacturers were obviously discrete. The ratio of a given chromatographic peak in the principal component was given by the load diagram of principal components, as shown in Fig. 5. The farther the distance from the y axis, the larger the contribution to PC1, e.g., peak 44 (KRCG), peak 42 (QCG) and peak 3 (PA); meanwhile, the farther the distance from the x axis, the larger the contribution to PC2, e.g., peak 3 (PA). The peak 44 (KRCG), peak 42 (QCG) and peak 3 (PA) were the main components which caused the difference between batches of GBP samples.

## Discussion

In order to fully reflect the quality of ginkgo leaf preparation, this experiment focused on various polar compounds. We analyzed step by step from low concentration organic phase to high concentration organic phase, the differences of various polar components were reflected more comprehensive. A novel reverse-phase liquid chromatography assay using diode array detection was developed for evaluating Ginkgo biloba preparations for quality based on a chromatographic fingerprint allowing the simultaneous assessment of eleven compounds, including four organic acids, six flavonol glycosides and one flavonoid aglycone.

Data analysis of the 51 batches of GBP samples suggested significant variations of the total contents of the 11 targets, demonstrating the quality differences of these GBP samples. There were significant differences in organic acids in particular.

The fingerprint analysis results of 51 batches of GBP samples showed that the similarity values of samples were low. Multivariate statistical analysis showed that samples could not be clustered by manufacturers. The possible reasons for the significant differences of batches were analyzed as below.

The raw material of GBP was ginkgo biloba extract, which was included in the filing management system in China. Ginkgo biloba preparation production companies can purchase the filed extract from other companies for production. As a result, the raw materials of Ginkgo biloba extract used for producing GBP in different manufacturers may come from the same manufacturer, and the raw materials of GBP produced by the same manufacturer can also come from different Ginkgo biloba extract manufacturers. Therefore, clustering of samples from different Ginkgo biloba preparation manufacturers was not obvious, and the GBP samples produced by the same manufacturer also had no obvious clustering trend.

The UPLC fingerprint is also analyzed by the assay proposed by the Chinese Pharmacopoeia 2020 edition [6], which is mainly focus on flavonol glycosides components, and the similarity valueswere greater than 0.9 (data not shown), indicating that the low similarity values were mainly caused by differences in organic acids. Moreover, in content determination data, great differences in the composition of organic acids were also found among various batches. The differences in organic acids may be due to different raw materials and production processes of Ginkgo biloba extract. The preparation method for Ginkgo biloba extract in the Chinese pharmacopoeia (2020 Edition) [6] is to extract with dilute ethanol, purify with macroporous adsorption resin, elute with water and different concentrations of ethanol in turn, and collect the eluent. It is speculated that differences in organic acid contents may be caused by the different washing volumes of water applied. It is also possible that retention of organic acids differs for different types of macroporous resin. Different manufacturers have divergent understanding of dilute ethanol. Each extraction manufacturer has own regulations and ethanol concentrations are 45–90%. The difference of processing parameters of different manufacturers may cause differences in organic acid contents.

## Conclusions

Here, a simple and reliable UPLC fingerprint was developed for GBP quality evaluation and simultaneous determination of its major bioactive components, including organic acids, flavonoid glycosides and free flavonoids, to comparatively assess the quality of Ginkgo biloba preparation (GBP) samples from different manufacturers. In this study, for the first time, the fingerprint contains not only flavonoids, but also organic acids, which more comprehensively reflects the sample quality and could benefit the quality control of Ginkgo biloba preparations. Data analysis of the 51 batches of GBP samples suggested that there are significant variations of the total contents of the 11 targets, demonstrating the quality differences of the GBP samples. There were significant differences in organic acids in particular. Chemometric analyses were performed for qualitatively evaluating GBP after the fingerprint analyses of GBP. However, different batches of representative specimens produced by different enterprises did not precisely cluster in HCA and PCA according to 59 commonly detected peaks.

Combining the novel chemical fingerprint and quantitative assessment revealed significant variations in the commercial products examined with regard to organic acids. Thus, this study provided a more comprehensive way for monitoring the quality consistency of Ginkgo biloba preparations.

## Data Availability

The datasets used and/or analyzed during the current study are available from the corresponding author on reasonable request.

## Abbreviations

TCM: Traditional chinese medicine
SA: Similarity analysis
HCA: Hierarchical clustering analysis
PCA: Principal component analysis
GbE: Ginkgo biloba leaf extract
RTs: Retention times
Pas: Peak areas
GA: Gallic acid
PA: Protocatechuic acid
pHA: P-Hydroxybenzoic acid
IR: Isorhamnetin 3-rutinoside
6-HKA: 6-Hydroxykynurenic acid
QDG: Quercetin 3-O-(2″, 6″-α-l-dirhamnopyranosyl)-β-d-glucoside
KR: Kaempferol-3-O-rutinoside
QCG: Quercetin-3-O-α-l-rhamnopyranocyl-2''-(6'''-p-coumaroyl)-β-d-glucoside
KRCG: Kaempferol-3-O-α-l-rhamnopyranocyl-2''-(6'''-p-coumaroyl)-β-d-glucoside
